# Childhood Hyperactivity, Physical Aggression and Criminality: A 19-Year Prospective Population-Based Study

**DOI:** 10.1371/journal.pone.0062594

**Published:** 2013-05-01

**Authors:** Jean-Baptiste Pingault, Sylvana M. Côté, Eric Lacourse, Cédric Galéra, Frank Vitaro, Richard E. Tremblay

**Affiliations:** 1 Research Unit on Children's Psychosocial Maladjustment, University of Montreal and Sainte–Justine Hospital, Montreal, Quebec, Canada; 2 International Laboratory for Child and Adolescent Mental Health Development, University of Montreal, Montreal, Quebec, Canada; 3 Department of Sociology, University of Montreal, Montreal, Quebec, Canada; 4 University of Bordeaux, INSERM U897, Charles Perrens Hospital, Child Psychiatry Department Bordeaux, France; 5 School of Public Health, Physiotherapy and Population Science, University College Dublin, Dublin, Ireland; 6 Departments of Pediatrics, Psychiatry and Psychology, University of Montreal, Montreal, Quebec, Canada; Universidade Federal do Acre (Federal University of Acre), Brazil

## Abstract

**Background:**

Research shows that children with Attention Deficit/Hyperactivity Disorder are at elevated risk of criminality. However, several issues still need to be addressed in order to verify whether hyperactivity in itself plays a role in the prediction of criminality. In particular, co-occurrence with other behaviors as well as the internal heterogeneity in ADHD symptoms (hyperactivity and inattention) should be taken into account. The aim of this study was to assess the unique and interactive contributions of hyperactivity to the development of criminality, whilst considering inattention, physical aggression and family adversity.

**Methodology/Principal Findings:**

We monitored the development of a population-based sample of kindergarten children (N = 2,741). Hyperactivity, inattention, and physical aggression were assessed annually between the ages of 6 and 12 years by mothers and teachers. Information on the presence, the age at first charge and the type of criminal charge was obtained from official records when the participants were aged 25 years. We used survival analysis models to predict the development of criminality in adolescence and adulthood: high childhood hyperactivity was highly predictive when bivariate analyses were used; however, with multivariate analyses, high hyperactivity was only marginally significant (Hazard Ratio: 1.38; 95% CI: 0.94–2.02). Sensitivity analyses revealed that hyperactivity was not a consistent predictor. High physical aggression was strongly predictive (Hazard Ratio: 3.44; 95% CI: 2.43–4.87) and its role was consistent in sensitivity analyses and for different types of crime. Inattention was not predictive of later criminality.

**Conclusions/Significance:**

Although the contribution of childhood hyperactivity to criminality may be detected in large samples using multi-informant longitudinal designs, our results show that it is not a strong predictor of later criminality. Crime prevention should instead target children with the highest levels of childhood physical aggression and family adversity.

## Introduction

Abundant evidence demonstrates that children diagnosed with Attention Deficit/Hyperactivity Disorder (ADHD) are at high risk of many long-term adverse outcomes, including criminality [1]–[5]. However, whether hyperactivity in itself plays a role in the prediction of criminality remains unclear, with mixed findings in clinical as well as population-based studies [1], [4]–[12]. Resolving this issue is important in order to: 1) clarify the role of hyperactivity in the developmental pathways leading to criminality; and 2) assess whether early symptoms of hyperactivity may be good targets for interventions aiming to prevent criminal behavior during adolescence and early adulthood.

In order to clarify the role of hyperactivity in the prediction of criminality, several limitations in the literature need to be addressed. First, the internal heterogeneity in ADHD symptoms needs to be acknowledged. Research suggests that symptoms of hyperactivity and inattention may have specific long-term consequences [10], [13]–[15] and aggregating them may obscure their specific contribution. Among the few studies that have distinguished between the two dimensions in regard to the prediction of criminality, some found a predominant role for hyperactivity [10], [16] while another reported the reverse [17]. Second, co-occuring externalized behaviors need to be taken into account in order to determine the specific contribution of hyperactivity relative to correlated behaviors (i.e., confounders) such as physical aggression [18]. Physical aggression during elementary school years is particularly important as a possible confounder for the following reasons: 1) physical aggression appears as early as hyperactivity during the preschool years [19]–[23]; 2) physical aggression is strongly correlated with hyperactivity in childhood [14], [22], [24]; 3) physical aggression is highly stable from early childhood to adulthood [25]–[27]; and 4) is eventually considered a criminal behavior if maintained during adolescence and early adulthood.

Third, developmental considerations may explain some discrepant results in the available literature: 1) variations in age at assessment of behavioral predictors may influence their predictive power; and 2) hyperactivity may contribute specifically to the early initiation of criminality. In particular, variations in age at behavioral assessments may have contributed to the discrepancy between the two large prospective population-based studies previously used to examine the association between hyperactivity and criminal records in adolescence and early adulthood. In one Finish study, teacher rated hyperactivity at 8 years predicted criminal records between 16 and 20 years [12]. Another study of females and males (mainly from rural counties of North Carolina) showed that ADHD symptoms rated by mothers and children were not associated with criminal records between 16 and 21 years of age [6]. The North Carolina study included any diagnosis for which the child met full diagnostic criteria by age 16 years. Hence, it is possible that assessments of hyperactivity in pre and early adolescence are less strongly related to criminality. The age at which the predictors of criminality are assessed is especially important for preventive intervention purposes.

Fourth, the role of hyperactivity might differ depending on the type of crimes [12], [16]. Fifth, few studies included a large number of females although the predictive role of hyperactivity may differ between sexes [28]–[30]. Sixth, some but not all studies included contextual variables such as family adversity. As shown by many studies, including the North Carolina and Finnish studies described above [6], [12], childhood family adversity is a good predictor of criminality in later years. It is also correlated with hyperactivity. We therefore included family adversity in our models.

Finally, we also tested for potential interaction effects between hyperactivity and inattention and between hyperactivity and physical aggression to test for potential synergetic effects [31]. Studies have suggested that individual characteristics such as hyperactivity could be more predictive of criminality when subjects have experienced elevated levels of adversity [32]. We therefore tested the interaction between hyperactivity and adversity as well as a triple interaction between hyperactivity, physical aggression and adversity.

The present study appears to be the largest and longest population-based, multiple informant, longitudinal study of females and males aimed to investigate the role of hyperactivity as a predictor of specific types of criminal charges during adolescence and early adulthood. Children, rated by both mothers and teachers on hyperactivity, inattention and physical aggression between the ages of 6 and 12 years, were followed until early adulthood to monitor involvement, the age of onset and the type of criminal behavior.

## Materials and Methods

### Ethics Statement

The study has been approved by the University of Montreal Ethics Committee. After complete description of the study, written consent was obtained from the mothers at each wave of data collection (including consent regarding teachers' reports).

### Participants

The 2,741 participants (1,398 boys) were attending kindergarten in Quebec's French-speaking public schools (Canada) between 1986 and 1988. Approximately two thirds of the participants (2,000) were representative of the kindergarten population, while close to one third (741) were selected to over sample those above the 80^th^ percentile of the Social Behavior Questionnaire (SBQ) disruptive scale [33]. The boys and girls were then assessed annually with the SBQ by teachers and mothers throughout their elementary school years. Criminal records were obtained when the subjects were 25 years old.

### Measures

#### Criminal records

Official Court records were available to assess criminality; they included all criminal charges (i.e. criminal records including non-violent only, drug-related only, violent only and mixed criminal charges) independently of conviction. A total of 401 participants (14.6%) had a criminal record. From these records, we obtained the participants' age at first infraction as well as the type of criminal charge.

#### Childhood behavior

Teachers assessed children's behaviors with the SBQ [33] between the ages of 6 and 12 years (a teacher taught only at one level so that the assessments were made by a different teacher each year). Mothers also assessed children yearly with the SBQ during this period. The SBQ is based upon the Children's Behavior Questionnaire [34] and the Preschool Behavior Questionnaire [35] which both demonstrated adequate psychometric properties. These results were replicated with the SBQ [33]. Furthermore, the SBQ was used in several large sample cohorts that documented its predictive validity on a range of adolescent and adult outcomes [24], [36]. Each item was rated on a 3-point scale (0 to 2) ranging from “never applies” to “frequently applies”. From age 6 to 12 years, four items were used to assess inattention: 1) Weak capacity for concentration, cannot maintain his/her attention for a long time on the same task 2) Easily distracted 3) Absentmindedness 4) Gives up easily (Cronbach's alphas for teachers between .85–.90; Cronbach's alphas for mothers between .71–.80). Two items were used to assess hyperactivity between the ages of 6 and 12 years: 1) Restless, runs about, or jumps up and down, does not keep still 2) Squirmy, fidgety child (alphas for teachers: .85–.89; alphas for mothers: .76–.79). Between the ages of 8 and 12 years, three additional items were available to assess impulsivity: 3) Jumps from one activity to another 4) Shouts to draw attention 5) Acts without thinking. We used this five-item measure of hyperactivity/impulsivity in sensitivity analyses (alphas for teachers: .83–.86; for mothers: .75–.76). Finally, between 6 and 12 years, three items were used to assess physical aggression: 1) Fights with other children 2) Bullies other children 3) Kicks, bites, or hits other children (alphas for teachers: .81–.88; for mothers: .60–.69).

#### Family Adversity Index

This index was based on information collected at the beginning of the study when the children were ending kindergarten. This index was based on the following indices: 1) family structure (intact or not intact), 2) parents' levels of education, 3) parents' occupational status [37] and 4) parents' age at the birth of the first child. Families at or below the 30th percentile on each of these indices (or a not intact family) were assigned a score of 1; remaining participants were coded zero. We then *averaged* these indices for each participant to obtain a family adversity score ranging from 0 to 1.

### Data analysis

#### Developmental trajectories

Data from multiple informants are considered more valid than data from a single informant [38]. To take into account both informants' assessments in a longitudinal design, we used developmental trajectory analyses. We estimated trajectories of inattention, hyperactivity, and physical aggression symptoms using k–means for longitudinal data [39], [40]. In this procedure, participants who are homogenous in their behavioral development are assigned to a given trajectory. In the present study, we employed a three-dimensional version of this procedure [41]: this procedure is original as it provides developmental trajectories for each behavior (e.g. inattention) relying on two types of informants instead of one.

**Figure 1:**
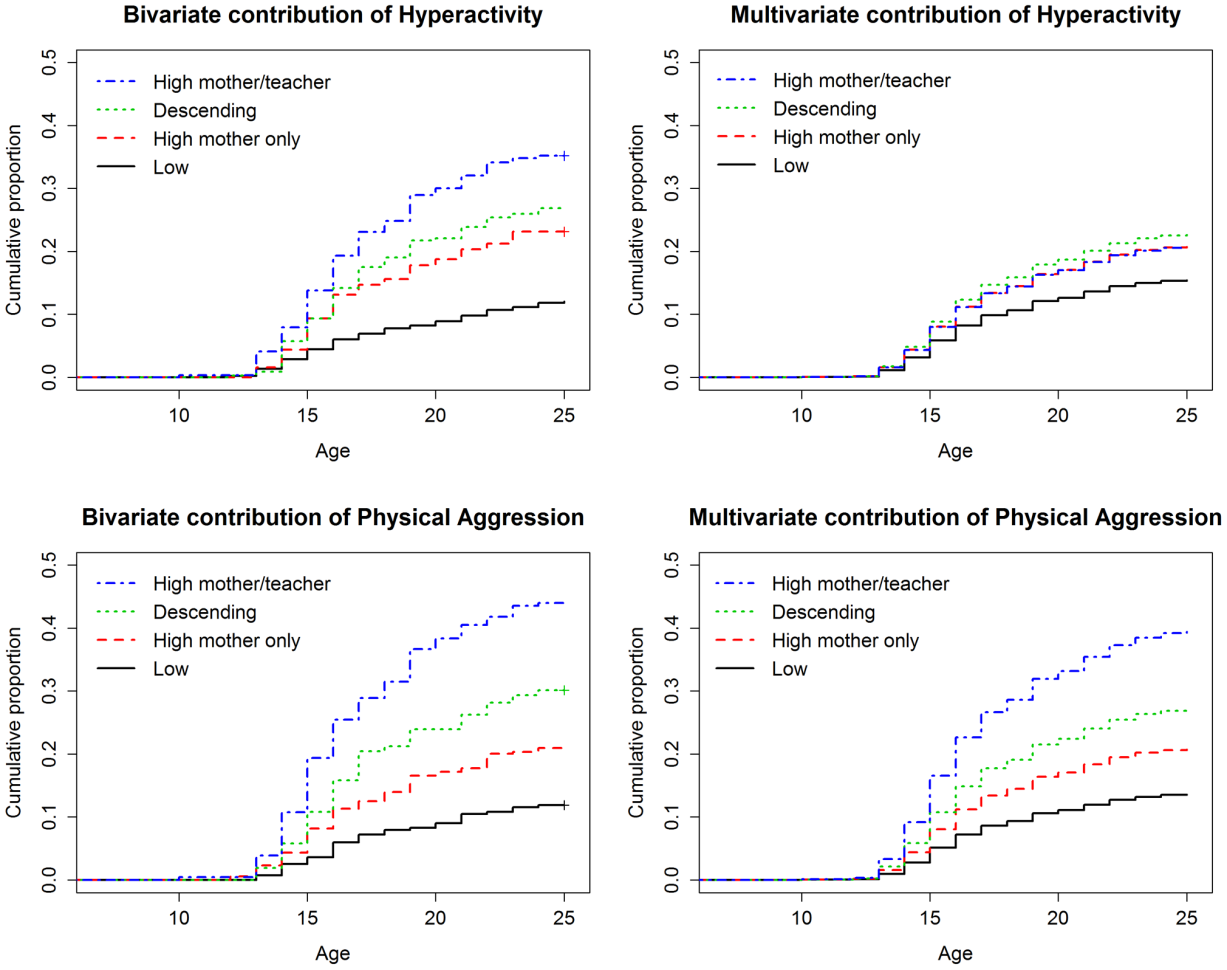
Survival Models: Contributions of Hyperactivity and Physical Aggression to the Development of Criminality in Males. The bivariate contributions are based on Kaplan-Meier plots. The adjusted contributions were plotted from multivariate Cox models. The values for covariates were: 1 for sex (i.e. male); mean adversity level; second trajectory (High mother only) for hyperactivity and physical aggression; low trajectory for inattention.

*Survival analysis* models the time it takes for events to occur (e.g. getting a criminal record). Since such events do not occur for all participants, two types of information are needed: whether an event occurred during the follow-up period (binary variable) and the time for the event to occur. When no event occurs during the follow-up period, the time variable is the duration of the follow-up. We conducted survival analyses by fitting a Cox regression with the age at first infraction as the time variable and the presence of a court record at the end of the follow-up period (25 years) as the event information. We thus obtained the cumulative proportion of events (inverse of the survival function) as a function of the age of the participants. When predictors were entered in the regression, the cumulative proportion was obtained for different groups of participants with different levels of risk (Figure 1 illustrates the cumulative proportion of participants with a criminal record separately for each of the hyperactivity or physical aggression trajectories). The behavioral trajectories, sex and family adversity served as predictors in the Cox regression.

#### Missing data

Trajectories were estimated for participants who had at least one mother assessment and one teacher assessment for each behavior. One participant did not satisfy this condition and was excluded from the analyses. Furthermore, eight of the 401 participants with a court record did not have information on age at first infraction. These participants were excluded from the survival analyses (repeating the analyses including these 8 participants with age at first infraction set to 25 years did not change the results). The family adversity index was missing for 150 participants (5.5%).We conducted a single imputation of the missing values based on the constituent variables of the index and behavioral characteristics of the child at age 6 years as assessed by teachers and mothers (later behaviors and court records were not used in the imputation) [42].

#### Complementary analyses

Analyses were conducted to assess whether the results were sensitive to: 1) the use of trajectory analysis: we used a different number of trajectories and we also averaged the yearly scores across the 7 years and used the average scores as predictors instead of trajectories; 2) the periods of assessment of childhood behaviors and criminality: we averaged children's behavioral scores across different developmental periods (i.e. 6–7 years and 8–12 years) and distinguished crimes committed during adolescence from crimes committed during early adulthood; 3) the over-sampling of disruptive children: we re-estimated the models without the children over-sampled for disruptive behaviors; 4) the informants: we examined mother and teacher rated behaviors separately; 5) the types of criminal charges (i.e. criminal records including non-violent only, drug-related only, violent only and mixed criminal charges). Further details are provided (see File S1, p.2).

## Results

### Behavioral trajectories

The proportion of participants in each trajectory is presented in Table 1 (first column). Additional figures (File S1, p.8. to p.14) allow the reader to 1) visualize the trajectories and the behavioral score levels within each trajectory according to mother and teacher ratings and 2) explore the three-dimensional trajectories in a dynamic fashion. We found two trajectories–high and low–for inattention. The four trajectories of hyperactivity and physical aggression followed a similar pattern: a “Low” trajectory; a “High mother only” trajectory with participants whose score was consistently among the sample highest scores according to their mother but amongst the lowest according to their teachers; a “Declining trajectory” with participants starting high and declining during elementary school; a “High mother/teacher” trajectory for a minority of participants who were rated constantly high by their mothers *and* their teachers. The process that led to the selection of a two trajectory model for inattention and a four trajectory model for hyperactivity and physical aggression is detailed in the Supporting Information (see File S1, p.8).

**Table 1:** Survival Models Predicting the Age at First Infraction based on Official Court Records.

	Court records (%)	Court records (Cox models)		
		uHR	aHR	95% CI
Inattention trajectories				
Low (57.6%)	9.8	-	-	-
High (42.4%)	21.2	2.24^***^	1.08	0.85–1.38
Hyperactivity trajectories				
Low (41.8%)	7.2	-	-	-
High mother only (25.9%)	13.5	1.93^***^	1.39^*^	1.01–1.90
Descending (18.1%)	23.0	3.39^***^	1.53^*^	1.09–2.16
High mother/teacher (14.2%)	28.0	4.33^***^	1.38^†^	0.94–2.02
Physical aggression				
Low (55.5%)	6.9	-	-	-
High mother only (21.9%)	15.2	2.28^***^	1.59^**^	1.18–2.15
Descending (13.1%)	25.6	4.00^***^	2.16^***^	1.54–3.03
High mother/teacher (9.5%)	43.4	7.72^***^	3.44^***^	2.43–4.87
Sex		-	-	-
Females (49.0%)	5.4			
Males (51.0%)	23.5	4.64^***^	3.05^***^	2.31–4.01
Family adversity				
Low (89.9%)	13.4	-	-	-
High (10.1%)	25.3	3.55^***^	2.40^***^	1.65–3.50

*Note*. The table presents the results of a Cox model (with robust variance) predicting the age at the first infraction documented in the court records. The first column shows the percentages of participants in each trajectory (e.g. 9.5% of the participants were classified in the High mother/teacher trajectory of physical aggression). The second column reports the percentage of events, i.e. whether one crime was recorded or not, irrespective of the age at which it was committed (e.g. of the 9.5% participants in the High mother/teacher trajectory of physical aggression, 43.4% had a criminal record by age 25 years). The last columns present unadjusted Hazard Ratios (uHR) as well as adjusted Hazard Ratios (aHR) based on the multivariate survival models. Low trajectories and Females are the contrast. Regarding adversity, we used the continuous variable in the analyses but, in order to better understand the data, we present in the second column the percentage of crimes in the highest decile (25.3%). ^***^p<.001; ^**^p<.01; ^*^p<.05; ^†^p<.10.

### Survival analyses

Table 1 (column 2) shows the proportion of court records in each behavioral trajectory. For instance, children who were classified in the low physical aggression trajectory were only 6.9% to have a criminal record by age 25 years. Conversely, children who were classified in the high mother/teacher trajectory of physical aggression were 43.4% to have a criminal record by age 25 years. Table 1 also presents unadjusted and adjusted Hazard Ratios corresponding to the contributions of behavioral trajectories as well as sex and adversity. All predictors, including hyperactivity, were significantly associated with criminality in bivariate analyses. In multivariate survival models of criminality: inattention was not a significant predictor anymore; hyperactivity trajectories had a small multivariate contribution which was significant for two trajectories (“High mother only” and “Descending” trajectories) but only marginally significant for the “High mother/teacher” trajectory; in contrast, physical aggression trajectories were all highly significant, e.g. for the “High mother/teacher” trajectory (aHR: 3.44; 95% CI: 2.43–4.87). Being male and living in a family with high levels of adversity also contributed significantly to the prediction of criminality in the multivariate survival analysis. Figure 1 plots the survival models, illustrating the contribution of hyperactivity and physical aggression trajectories to the cumulative proportion of people committing a first infraction. We also present the same graphs for inattention trajectories, sex and family adversity (see File S1, p.7).

One requirement of the Cox model is the fulfillment of the proportional hazards assumption [43], meaning that the contribution of a predictor has to be constant over time. This assumption was verified for nearly all predictors so that their effect was constant over time (e.g. hyperactivity did not contribute more during adolescence than in early adulthood). The only exception was sex: the bivariate figure indicates that the curve for females flattens after adolescence as very few additional events occur (see File S1, p.7). Reevaluations of the model with a restricted follow-up [43] demonstrated that, until 22 years, the proportional hazards assumption was not violated. However, it was violated when the follow-up included ages from 23 years and onward: The gap between males and females widened after adolescence and, thus, the effect of sex was not constant. No interaction between behavioral variables, sex and adversity was significant. Furthermore, we found no evidence of a synergetic effect between behavioral variables (i.e. no significant positive interactions) or an interaction effect involving physical aggression, hyperactivity and adversity.

### Complementary analyses

First, we verified whether the number of trajectories for each behavior influenced the results. When two trajectories instead of four were used for hyperactivity and physical aggression (i.e. the same number as inattention), physical aggression remained a very significant predictor of criminality whereas hyperactivity was not significant anymore. Table S1 (in File S1, p.3) presents the results based on average scores instead of trajectories (see *Method section*). To summarize, the contribution of physical aggression was very consistent: it remained significant irrespective of the period of behavioral assessment (6–7 or 8–12 years), for crimes in adolescence and adulthood and for both informants. Hyperactivity was significant in only one model: Mother rated hyperactivity/impulsivity at 8–12 years predicted adult criminality. Restricting the analyses to the random sample (N = 2000, see Table S2 in File S1, p.4) did not change the results. Finally, further details on the analyses regarding specific types of criminal charges for males are presented in the Supporting Information: see File S1, p.2 for details on the classification and the proportion of criminal records for each type of criminal charge; and see the results in Table S3 (Teachers' ratings, in File S1, p.5) and Table S4 (Mothers' ratings, in File S1, p.6). To summarize, physical aggression systematically predicted non-violent only and mixed types of crimes, in adolescence and adulthood, irrespective of the age at behavioral assessment (i.e. 6–7 or 8–12 years), and for both informants. Despite the lower prevalence of violent only crimes, physical aggression was also predictive in most models. Childhood behaviors did not predict drug-related only crimes as well as they predicted other types of crimes; notably, physical aggression was predictive in only one model and no consistent role was found for hyperactivity or inattention.

## Discussion

The purpose of this study was to clarify the contribution of childhood hyperactivity to criminality in a large prospective population-based study. In bivariate analyses, hyperactivity trajectories were a strong predictor of criminality. However, after having controlled for family adversity as well as trajectories of inattention and physical aggression, only two out of three childhood hyperactivity trajectories made a small significant contribution to the prediction of criminality. Furthermore, this contribution of hyperactivity was not consistent in sensitivity analyses. Conversely, physical aggression was the most important and consistent behavioral predictor of criminality. It should be noted that participants in the high mother/teacher trajectory of physical aggression, while constituting 9.5% of the sample, represented 28.2% of all the participants with a criminal record. In addition, they represented 45.9% of all recorded criminal charges and 57.4% of the violent ones. Therefore, participants in this high trajectory of physical aggression are not only more likely to have a criminal record but, when they have one, to have more criminal charges.

### Behaviors' specific contributions

In our study, hyperactivity seemed to be more predictive than inattention as it was found in two other studies that distinguished between these two dimensions [10], [16]. However, its role was rather inconsistent. Taking into account correlated behaviors was essential in assessing the contribution of hyperactivity. However, there was a risk of over-controlling for behaviors that may develop as a consequence of hyperactivity. For instance, there is some evidence that childhood hyperactivity fosters the development of later externalizing disorders, in particular conduct disorder, which in turn predicts adolescent and adult criminality [4], [9], [10], [44]. Controlling for such later developing potential mediators (e.g. violation status symptoms in conduct disorder) would reduce the contribution of hyperactivity and unduly minimize its role. However, these putative mediators or some of their constitutive symptoms (e.g. aggressive symptoms in conduct disorder) may not be mediators but correlates of hyperactivity. For example, in the present study, hyperactivity was not anterior to physical aggression and previous studies have shown that physical aggression appears as early as hyperactivity and peaks during the preschool years [19], [21]–[23]. Therefore, as a mediator is supposed to follow the predictor [45], physical aggression is not likely to be a mediator of the contribution of hyperactivity to criminality and was thus introduced in the models at the same level as hyperactivity. Although further research is needed to clarify the temporal sequence in the development of physical aggression and hyperactivity during (early) childhood, our results clearly support the notion that physical aggression needs to be taken into account when trying to understand the developmental impact of hyperactivity on later criminality. Our finding that hyperactivity did not predict adult criminality once its overlap with physical aggression was accounted for suggests that the positive association between hyperactivity during the elementary school years and criminal behavior observed in previous studies might be largely explained by the association between hyperactivity and physical aggression. Finally, we found no evidence of a synergetic effect between hyperactivity and inattention or physical aggression.

### Developmental issues

We hypothesized that the divergent results regarding the role of hyperactivity in the two previous large prospective population studies mentioned in the introduction [6], [12] could be due in part to the difference in age when hyperactivity was assessed: a significant role of hyperactivity was found in the study with earlier behavioral assessments–8 years old [12]. Given the decrease in the frequency of hyperactivity symptoms with age [14], [21], [46] it may have been possible that assessments of hyperactivity in pre and early adolescence could be less strongly related to criminality than earlier assessments of hyperactivity. However, our results did not confirm this hypothesis because assessing hyperactivity early (6–7 years) or later (8–12 years) did not change its predictive power. Finally, analyses with survival models indicated that there was no evidence to suggest that the role of childhood hyperactivity changed over time (e.g. that childhood hyperactivity was more predictive of criminality occurring in adolescence rather than in adulthood).

### Types of crimes

Previous studies suggested that hyperactivity could contribute more to drug-related and/or to non-violent crimes [1], [12], [16], [23]. In the present study, hyperactivity did not contribute at all to non-violent or mixed crimes whereas physical aggression was a consistent predictor across models. Violent-only crimes were less prevalent, but physical aggression was also significant in most models whereas hyperactivity was not. We found no consistent predictor for drug-related only crimes, so we cannot confirm or infirm a specific role for hyperactivity in this case.

### Sex and family adversity

Very few studies of the association between hyperactivity and criminality included female participants [6], [9], [16]. To our knowledge the present study includes the largest number of females to test the association between hyperactivity and criminality from kindergarten to adulthood. As expected sex was a strong predictor [6], [16]. It should be noted that its effect was not constant over time: as the new occurrences of charges decreased more in females after adolescence, the gap between the sexes widened at that age. We also tested whether the predictors of criminality would be different among females by examining the interactions between sex and the other predictors. None were significant. Family adversity was a strong predictor in itself as in previous studies but we detected no interaction with hyperactivity.

### Limitations

The use of court records may have avoided two potential biases in this study, the first of them being attrition because court records were available for all participants. Second, adolescent and young adults with ADHD symptoms have been shown to under-report their own delinquent acts and be inconsistent in their reporting, which could lead to a biased estimation of the effect of inattention and hyperactivity [47]. However, a limitation of court records is that they capture only a restricted amount of crimes; the use of several informants would have allowed us to verify whether our results were sensitive to the type of informant for the outcome as we did for the behavioral predictors. The low prevalence of criminal records often raises a power issue in longitudinal studies. This is why we used a large population sample. However, statistical power may have been an issue in some sensitivity analyses, i.e. the prediction of males' less frequent types of criminal records–violent and drug-related only criminal records–although physical aggression was significant in most models predicting violent only criminal records. Finally, inattention, hyperactivity, and physical aggression were measured by a well-validated questionnaire, although it does not assess all aspects of DSM inattention and hyperactivity.

### Conclusions and implications for prevention

This study is unique in that it used a large population sample of female and male kindergarten children, with annual teacher and mother rated behaviors over 7 years, in addition to a 19 year follow-up. Official records of criminal charges were available for all participants during both adolescence and early adulthood. We explored a number of potential issues that could have prevented adequate assessment of hyperactivity's contribution to criminality. We found that two hyperactivity trajectories, based on two informants and 7 years of assessment were predictive in survival analyses modeling the occurrences of crimes until 25 years of age. However, the role of hyperactivity was not true for all trajectories and was not verified consistently in sensitivity analyses. To conclude, although the contribution of childhood hyperactivity to criminality might be detected in large samples with strong multi-informant longitudinal designs, it is not, by far, the best predictor of later criminality.

Consequently, childhood hyperactivity is not likely to be the best focus for preventive interventions of criminal behavior. Our results suggest instead focusing on childhood physical aggression. Finally, the magnitude of the effect is to be stressed: addressing efficiently physical aggression and family adversity related issues in childhood may contribute to a substantial reduction in the number of people with criminal records and an even more substantial reduction of the total number of criminal charges and, in particular, violent ones.

## Supporting Information

File S1 **In the Supporting Information File S1, supplemental tables regarding the complementary analyses are provided.** Additional figures concerning the contributions of inattention and family adversity (plotted as in Figure 1 in the manuscript) as well as sex are presented. Finally, the rationale for the selection of the trajectories is given, accompanied by two dimensional as well as three dimensional dynamic representations of the trajectories, which can be manipulated by the viewer. An Index is provided on the first page of the File S1.
(PDF)

## References

[1] Barkley RA, Fischer M, Smallish L, Fletcher K (2004) Young adult follow-up of hyperactive children: Antisocial activities and drug use. J Child Psychol Psychiatry 45: 195–211 doi:10.1111/j.1469-7610.2004.00214.x. 14982236 10.1111/j.1469-7610.2004.00214.x

[2] Dalteg A, Levander S (1998) Twelve thousand crimes by 75 boys: A 20-year follow-up study of childhood hyperactivity. J Forensic Psychiatry 9: 39–57 doi:10.1080/09585189808402178.

[3] Mannuzza S, Klein RG, Konig PH, Giampino TL (1989) Hyperactive boys almost grown up: IV. Criminality and its relationship to psychiatric status. Arch Gen Psychiatry 46: 1073–1079 doi:10.1001/archpsyc.1989.01810120015004. 2589922 10.1001/archpsyc.1989.01810120015004

[4] Mannuzza S, Klein RG, Moulton III JL (2008) Lifetime criminality among boys with attention deficit hyperactivity disorder: A prospective follow-up study into adulthood using official arrest records. Psychiatry Res 160: 237–246 doi:10.1016/j.psychres.2007.11.003. 18707766 10.1016/j.psychres.2007.11.003 PMC2581455

[5] Rasmussen P, Gillberg C (2000) Natural outcome of ADHD with developmental coordination disorder at age 22 years: A controlled, longitudinal, community-based study. J Am Acad Child Adolesc Psychiatry 39: 1424–1431 doi:10.1097/00004583-200011000-00017. 11068898 10.1097/00004583-200011000-00017

[6] Copeland WE, Miller-Johnson S, Keeler G, Angold A, Costello EJ (2007) Childhood psychiatric disorders and young adult crime: A prospective, population-based study. American Journal of Psychiatry 164: 1668–1675 doi:10.1176/appi.ajp.2007.06122026. 17974931 10.1176/appi.ajp.2007.06122026

[7] Fletcher J, Wolfe B (2009) Long-term consequences of childhood ADHD on criminal activities. J Ment Health Policy Econ 12: 119–138 doi:10.2139/ssrn.1489147. 19996475 PMC3398051

[8] Lacourse E, Baillargeon R, Dupéré V, Vitaro F, Romano E, et al (2010) Two-year predictive validity of conduct disorder subtypes in early adolescence: A latent class analysis of a Canadian longitudinal sample. J Child Psychol Psychiatry 51: 1386–1394 doi:10.1111/j.1469-7610.2010.02291.x. 20695929 10.1111/j.1469-7610.2010.02291.x

[9] Mordre M, Groholt B, Kjelsberg E, Sandstad B, Myhre AM (2011) The impact of ADHD and conduct disorder in childhood on adult delinquency: A 30 years follow-up study using official crime records. BMC Psychiatry 11: 57 doi:10.1186/1471-244X-11-57. 21481227 10.1186/1471-244X-11-57 PMC3082292

[10] Pardini DA, Fite PJ (2010) Symptoms of Conduct Disorder, Oppositional Defiant Disorder, Attention-Deficit/Hyperactivity Disorder, and callous-unemotional traits as unique predictors of psychosocial maladjustment in boys: Advancing an evidence base for DSM-V. J Am Acad Child Adolesc Psychiatry 49: 1134–1144 doi:10.1016/j.jaac.2010.07.010. 20970701 10.1016/j.jaac.2010.07.010 PMC3064931

[11] Satterfield JH, Faller KJ, Crinella FM, Schell AM, Swanson JM, et al (2007) A 30-year prospective follow-up study of hyperactive boys with conduct problems: Adult criminality. J Am Acad Child Adolesc Psychiatry 46: 601–610 doi:10.1097/chi.0b013e318033ff59. 17450051 10.1097/chi.0b013e318033ff59

[12] Sourander A, Elonheimo H, Niemela S, Nuutila A-M, Helenius H, et al (2006) Childhood predictors of male criminality: A prospective population-based follow-up study from age 8 to late adolescence. J Am Acad Child Adolesc Psychiatry 45: 578–586 doi:10.1097/01.chi0000205699.58626.b5. 16670652 10.1097/01.chi0000205699.58626.b5

[13] Lee SS, Hinshaw SP (2004) Severity of adolescent delinquency among boys with and without attention deficit hyperactivity disorder: Predictions from early antisocial behavior and peer status. J Clin Child Adolesc Psychol 33: 705–716 doi:10.1207/s15374424jccp3304_6. 15498738 10.1207/s15374424jccp3304_6

[14] Pingault J-B, Tremblay RE, Vitaro F, Carbonneau R, Genolini C, et al (2011) Childhood trajectories of inattention and hyperactivity and prediction of educational attainment in early adulthood: A 16-year longitudinal population-based study. Am J Psychiatry 168: 1164–1170 doi:10.1176/appi.ajp.2011.10121732. 21799065 10.1176/appi.ajp.2011.10121732

[15] Pingault J-B, Côté SM, Galéra C, Genolini C, Falissard B, et al. (2013) Childhood trajectories of inattention, hyperactivity and oppositional behaviors and prediction of substance abuse/dependence: A 15-year longitudinal population-based study. Mol Psychiatry. Available:http://www.ncbi.nlm.nih.gov/pubmed/22733124. Accessed 15 July 2012.doi: 10.1038/mp.2012.87. (In Press) 10.1038/mp.2012.87 PMC3954095 22733124

[16] Babinski LM, Hartsough CS, Lambert NM (1999) Childhood conduct problems, hyperactivity-impulsivity, and inattention as predictors of adult criminal activity. J Child Psychol Psychiatry 40: 347–355 doi:10.1111/1469-7610.00452. 10190336

[17] Eklund JM, Klinteberg BA (2003) Childhood behaviour as related to subsequent drinking offences and violent offending: A prospective study of 11- to 14-year-old youths into their fourth decade. Crim Behav Ment Health 13: 294–309 doi:10.1002/cbm.552. 14654865 10.1002/cbm.552

[18] Boots DP, Wareham J (2010) Does controlling for comorbidity matter? DSM-oriented scales and violent offending in Chicago youth. Aggress Behav 36: 141–157 doi:10.1002/ab.20338. 20127817 10.1002/ab.20338

[19] Côté S, Vaillancourt T, LeBlanc JC, Nagin DS, Tremblay RE (2006) The development of physical aggression from toddlerhood to pre-adolescence: A nation wide longitudinal study of Canadian children. J Abnorm Child Psychol 34: 68–82 doi:10.1007/s10802-005-9001-z. 10.1007/s10802-005-9001-z 16565888

[20] NICHD (2004) Trajectories of physical aggression from toddlerhood to middle childhood: Predictors, correlates, and outcomes. Monogr Soc Res Child Dev 69 : vii, 1–129. doi:10.1111/j.0037-976X.2004.00312.x. 10.1111/j.0037-976x.2004.00312.x 15667346

[21] Galéra C, Côté SM, Bouvard MP, Pingault J-B, Melchior M, et al (2011) Early risk factors for hyperactivity-impulsivity and inattention trajectories from age 17 months to 8 years. Arch Gen Psychiatry 68: 1267–1275 doi:10.1001/archgenpsychiatry.2011.138. 22147844 10.1001/archgenpsychiatry.2011.138

[22] Huijbregts SCJ, Séguin JR, Zoccolillo M, Boivin M, Tremblay RE (2007) Associations of maternal prenatal smoking with early childhood physical aggression, hyperactivity-impulsivity, and their co-occurrence. J Abnorm Child Psychol 35: 203–215 doi:10.1007/s10802-006-9073-4. 17294130 10.1007/s10802-006-9073-4 PMC1915590

[23] Stevenson J, Goodman R (2001) Association between behaviour at age 3 years and adult criminality. Br J Psychiatry 179: 197–202 doi:10.1192/bjp.179.3.197. 11532795 10.1192/bjp.179.3.197

[24] Fontaine N, Carbonneau R, Barker ED, Vitaro F, Hébert M, et al (2008) Girls' hyperactivity and physical aggression during childhood and adjustment problems in early adulthood: A 15-year longitudinal study. Arch Gen Psychiatry 65: 320–328 doi:10.1001/archgenpsychiatry.2007.41. 18316678 10.1001/archgenpsychiatry.2007.41

[25] Eron L (1987) The development of aggressive behavior from the perspective of developing behaviorism. Am Psychol 42: 435–442. 3605821 10.1037//0003-066x.42.5.435

[26] Olweus D (1979) Stability of aggressive reaction patterns in males: A review. Psychol Bull 86: 852–875. 482487

[27] Tremblay RE (2000) The development of aggressive behaviour during childhood: What have we learned in the past century? Int J Behav Dev 24: 129–141.

[28] Biederman J, Petty CR, Monuteaux MC, Fried R, Byrne D, et al (2010) Adult psychiatric outcomes of girls with ADHD: 11-year follow-up in a longitudinal case-control study. Am J Psychiatry 167: 409–417 doi:10.1176/appi.ajp.2009.09050736. 20080984 10.1176/appi.ajp.2009.09050736

[29] Lahey BB, McBurnett K, Loeber R (2000) Are attention-deficit/hyperactivity disorder and oppositional defiant disorder developmental precursors to conduct disorder. Handbook of Developmental Psychopathology. New York: Kluwer Academic/Plenum Publishers.

[30] Lee SS, Hinshaw SP (2006) Predictors of adolescent functioning in girls with attention deficit hyperactivity disorder (ADHD): The role of childhood ADHD, conduct problems, and peer status. J Clin Child Adolesc Psychol 35: 356–368 doi:10.1207/s15374424jccp3503_2. 16836474 10.1207/s15374424jccp3503_2 PMC2930194

[31] Schonberg MA, Shaw DS (2007) Do the predictors of child conduct problems vary by high- and low-levels of socioeconomic and neighborhood risk? Clin Child Fam Psychol Rev 10: 101–136 doi:10.1007/s10567-007-0018-4. 17394059 10.1007/s10567-007-0018-4

[32] Lynam DR, Caspi A, Moffitt TE, Wikström PO, Loeber R, et al (2000) The interaction between impulsivity and neighborhood context on offending: The effects of impulsivity are stronger in poorer neighborhoods. J Abnorm Psychol 109: 563–574. 11195980 10.1037//0021-843x.109.4.563

[33] Tremblay RE, Loeber R, Gagnon C, Charlebois P, Larivée S, et al (1991) Disruptive boys with stable and unstable high fighting behavior patterns during junior elementary school. J Abnorm Child Psychol 19: 285–300. 1865046 10.1007/BF00911232

[34] Rutter M (1967) A children's behaviour questionnaire for completion by teachers: Preliminary findings. J Child Psychol Psychiatry 8: 1–11. 6033260 10.1111/j.1469-7610.1967.tb02175.x

[35] Behar L, Strinfield S (1974) A behavior rating scale for the preschool child. Dev Psychol 10: 601–610.

[36] Nagin DS, Tremblay RE (1999) Trajectories of boys' physical aggression, opposition, and hyperactivity on the path to physically violent and nonviolent juvenile delinquency. Child Dev 70: 1181–1196. 10546339 10.1111/1467-8624.00086

[37] Blishen BR, Carroll WK, Moore C (1987) The 1982 socioeconomic index for occupations in Canada. Can Rev Soc Anthrop 24: 465–488.

[38] Achenbach TM, McConaughy SH, Howell CT (1987) Child/adolescent behavioral and emotional problems: Implications of cross-informant correlations for situational specificity. Psychol Bull 101: 213–232. 3562706

[39] Genolini C, Falissard B (2010) KmL: K-means for longitudinal data. Comput Stat 25: 317–328 doi:10.1007/s00180-009-0178-4.

[40] Genolini C, Falissard B (2011) KmL: A package to cluster longitudinal data. Comput Methods Programs Biomed 104: e112–121 doi:10.1016/j.cmpb.2011.05.008. 21708413 10.1016/j.cmpb.2011.05.008

[41] Genolini C, Pingault JB, Driss T, Côté S, Tremblay RE, et al (2013) KmL3D: a non-parametric algorithm for clustering joint trajectories. Comput Methods Programs Biomed 109: 104–111 doi:10.1016/j.cmpb.2012.08.016. 23127283 10.1016/j.cmpb.2012.08.016

[42] Van Buuren S, Groothuis-Oudshoorn K (2011) mice: Multivariate Imputation by Chained Equations in R. J Stat Softw 45: 1–67.

[43] Martinussen T, Scheike TH (2006) Dynamic regression models for survival data. Springer 470 p.

[44] Simonoff E, Elander J, Holmshaw J, Pickles A, Murray R, et al (2004) Predictors of antisocial personality. Continuities from childhood to adult life. Br J Psychiatry 184: 118–127 doi:10.1192/bjp.184.2.118. 14754823 10.1192/bjp.184.2.118

[45] Essex MJ, Kraemer HC, Armstrong JM, Boyce WT, Goldsmith HH, et al (2006) Exploring risk factors for the emergence of children's mental health problems. Arch Gen Psychiatry 63: 1246–1256 doi:10.1001/archpsyc.63.11.1246. 17088505 10.1001/archpsyc.63.11.1246

[46] Biederman J (2005) Attention-deficit/hyperactivity disorder: A selective overview. Biol Psychiatry 57: 1215–1220 doi:10.1016/j.biopsych.2004.10.020. 15949990 10.1016/j.biopsych.2004.10.020

[47] Sibley MH, Pelham WE, Molina BSG, Waschbusch DA, Gnagy EM, et al (2010) Inconsistent self-report of delinquency by adolescents and young adults with ADHD. J Abnorm Child Psychol 38: 645–656 doi:10.1007/s10802-010-9404-3. 20309624 10.1007/s10802-010-9404-3 PMC2918231

