# Supplementary material for: Childhood Hyperactivity, Physical Aggression and Criminality: A 19-Year Prospective Population-Based Study
Source: PLoS One. 2013 May 1;8(5):e62594. doi: 10.1371/journal.pone.0062594 (PMC3641049; doi:10.1371/journal.pone.0062594)
Supplement: File S1 — In the Supporting Information File S1, supplemental tables regarding the complementary analyses are provided. Additional figures concerning the contributions of inattention and family adversity (plotted as in Figure 1 in the manuscript) as well as sex are presented. Finally, the rationale for the selection of the trajectories is given, accompanied by two dimensional as well as three dimensional dynamic representations of the trajectories, which can be manipulated by the viewer. An Index is provided on the first page of the File S1. (PDF) [file pone.0062594.s001.pdf]

Supporting Information, File S1

Index

- Complementary analyses: procedures and results, *p.2.*
- Table S1: Prediction of Court Records with Averaged Behaviors, *p.3.*
- Table S2: Prediction of Court Records with Averaged Behaviors, Random Sample, *p.4.*
- Table S3: Prediction of Males' Non-Violent, Drug-related, Violent and Mixed Court Records (Teachers' ratings), *p.5.*
- Table S4: Prediction of Males' Non-Violent, Drug-related, Violent and Mixed Court Records (Mothers' ratings), *p.6.*
- Figures: survival graphs of inattention, family adversity and sex, *p.7.*
- Selection and representation of trajectories, *p.8.*
- Trajectories of inattention (two dimensions), *p.9.*
- Trajectories of inattention (three dimensions), *p.10.*
- Trajectories of hyperactivity (two dimensions), *p.11.*
- Trajectories of hyperactivity (three dimensions), *p.12.*
- Trajectories of physical aggression (two dimensions), *p.13.*
- Trajectories of physical aggression (three dimensions), *p.14.*

Complementary analyses: procedures

To verify whether the use of trajectory analyses would influence the results, we used averaged behavioral scores. As mentioned in the introduction, we wanted to verify whether the results would be affected by the time of assessments of both childhood behaviors and criminal outcomes (i.e. adolescent versus adult criminal records). We thus averaged the behavioral scores for two periods: 6-7 years; and 8-12 years. The 8-12 years averaged scores included the assessment of hyperactivity/impulsivity with 5 items (see Measures) instead of 2. We also recoded the presence of a criminal record for two age periods: before18 years (age of legal majority) and from 18 to 25 years. Criminal records from 18 to 25 years did not take into account any crime committed before age 18 years. Logistic regressions were used to estimate the contribution of these two sets of averaged scores to adolescent and adult criminal records. Mothers' and teachers' ratings were used separately. In order to verify whether the results were sensitive to the inclusion of participants who were selected because they had SBQ disruptive scores above the 80th percentile at the first assessment, we conducted the same analyses restricted to the random sample (N = 2000). Finally, we further analyzed specific kind of crimes with a categorical outcome variable in males (not enough crimes were committed by women to analyze each category separately). As in Sourander et al. (reference in the manuscript) we classified the criminal offences as non-violent, violent, and drug related but, unlike these authors, our categories were mutually exclusive (i.e., each participant belonged to only one category). The categories were coded: 0 when no criminal record was present; 1 for non-violent crimes only, i.e. one or more non-violent crimes (excluding cases with violent and drug-related crimes); 2 for drug-related only crimes, i.e. one or more drug-related crimes (excluding cases with violent and other non-violent crimes); 3 for violent crimes only (excluding non-violent and drug-related crimes); and 4 for a mixed category including court records with 2 or 3 types of crimes.

Complementary analyses: results

An overview of the results of the complementary analyses can be found in the manuscript. Table S1 presents sensitivity analyses which replicate the findings presented in the manuscript with averaged teacher and mother rated behaviors between 6-7 years and 8-12 years, predicting adolescent and adulthood criminality. Table S2 presents the same analyses restricted to the random sample, i.e. omitting the over-sampled disruptive children.

Tables S3 (Teachers' ratings) and S4 (Mothers' ratings) present the results for specific types of crimes in male participants (N = 1398). Violent criminal offences included offences with weapons or without weapons (e.g. homicide, assault). Non-violent criminal offences included all criminal offences not classified as violent or drug-related (e.g. property offences, motor vehicle-related offences, other criminal code offences such as prostitution). Drug-related crimes included all offences linked to drug possession or drug-trafficking. Out of 212 adolescent male participants with a criminal record, 110 had non-violent only, 22 drug-related, 20 violent and 60 mixed crimes. Out of 185 adult male participants with a criminal record, 106 had non-violent, 19 drug-related, 15 violent and 45 mixed crimes.

Table S1: Prediction of Court Records with Averaged Behaviors

	Teachers		Mothers	
	OR	95% CI	OR	95% CI
Adolescence				
6-7 years				
Inattention	1.04	0.97-1.11	1.02	0.94-1.10
Hyperactivity[1]	1.03	0.90-1.18	1.06	0.94-1.20
Physical aggression	1.30***	1.17-1.44	1.30***	1.16-1.45
Family adversity[2]	3.65***	2.26-5.88	3.75***	2.32-6.08
Sex	3.13***	2.28-4.36	3.48***	2.55-4.82
8-12 years				
Inattention	1.09*	1.00-1.18	1.02	0.93-1.12
Hyperactivity/Impulsivity[1]	1.01	0.92-1.11	1.07	0.98-1.17
Physical aggression	1.52***	1.32-1.75	1.33***	1.16-1.54
Family adversity[2]	3.35***	2.06-5.45	3.64***	2.21-6.00
Sex	2.67***	1.93-3.75	3.24***	2.34-4.53
Adulthood				
6-7 years				
Inattention	0.99	0.92-1.07	1.05	0.96-1.14
Hyperactivity[1]	0.96	0.83-1.12	1.03	0.89-1.18
Physical aggression	1.44***	1.29-1.61	1.26***	1.11-1.43
Family adversity[2]	2.70***	1.58-4.61	2.69***	1.57-4.62
Sex	5.68***	3.75-8.93	6.36***	4.23-9.95
8-12 years				
Inattention	1.08†	0.99-1.18	1.05	0.95-1.16
Hyperactivity/Impulsivity[1]	1.04	0.94-1.14	1.10*	1.00-1.21
Physical aggression	1.35***	1.16-1.57	1.26**	1.08-1.47
Family adversity[2]	2.54***	1.48-4.36	3.19***	1.82-5.56
Sex	5.23***	3.44-8.25	6.10***	4.01-9.68

Note. The table presents Odds ratios (OR) and 95% Confidence intervals (CI) of logistic regressions predicting criminal records. Teachers' ratings were available for all participants at 6-7 years (N = 2741) and missing for 28 at age 8-12 years (N = 2713). Mothers' ratings were missing for 10 participants at age 6-7 (N = 2731) and 180 at age 8-12 (N = 2561). [1]Assessments of hyperactivity at 6-7 years were based on two items; at 8-12 years, 5 items were available for hyperactivity/impulsivity (see *Method* section of the manuscript). [2]The family adversity index was identical in models at 6-7 years and models at 8-12 years; it was based on data collected when the child was 6 years and we used the imputed variable, see the *Method* section of the manuscript. ***p.<.001; **p.<.01; *p.<.05; †p.<.10.

Table S2: Prediction of Court Records with Averaged Behaviors, Random sample

	Teachers		Mothers	
	OR	95% CI	OR	95% CI
Adolescence				
6-7 years				
Inattention	1.06	0.98-1.16	1.05	0.95-1.16
Hyperactivity[1]	0.98	0.82-1.16	1.00	0.86-1.16
Physical aggression	1.40***	1.22-1.61	1.36***	1.17-1.57
Family adversity[2]	2.91***	1.59-5.30	3.32***	1.82-6.05
Sex	3.17***	2.16-4.77	3.44***	2.35-5.15
8-12 years				
Inattention	1.11*	1.01-1.23	1.04	0.93-1.16
Hyperactivity/Impulsivity[1]	1.00	0.88-1.13	1.10	0.99-1.22
Physical aggression	1.67***	1.38-2.02	1.29**	1.06-1.56
Family adversity[2]	2.66**	1.44-4.90	3.38***	1.82-6.26
Sex	2.63***	1.77-4.00	3.38***	2.28-5.14
Adulthood				
6-7 years				
Inattention	1.01	0.92-1.11	1.10†	0.98-1.22
Hyperactivity[1]	0.97	0.79-1.17	1.05	0.89-1.25
Physical aggression	1.47***	1.27-1.70	1.25**	1.06-1.47
Family adversity[2]	3.21***	1.64-6.27	3.44***	1.75-6.70
Sex	6.92***	4.08-12.56	7.45***	4.41-13.5
8-12 years				
Inattention	1.12*	1.00-1.24	1.07	0.94-1.21
Hyperactivity/Impulsivity[1]	1.06	0.93-1.2	1.14*	1.02-1.29
Physical aggression	1.43***	1.16-1.75	1.28*	1.04-1.57
Family adversity[2]	2.81**	1.42-5.52	3.91***	1.94-7.84
Sex	5.90***	3.45-10.78	6.85***	4.01-12.51

Note. The table presents Odds ratios (OR) and 95% Confidence intervals (CI) of logistic regressions predicting criminal records. Teachers' ratings were available for all participants at 6-7 years (N = 2000) and missing for 18 at age 8-12 years (N = 1982). Mothers' ratings were missing for 7 participants at age 6-7 (N = 1993) and 108 at age 8-12 (N = 1892). [1]Assessments of hyperactivity at 6-7 years were based on two items; at 8-12 years, 5 items were available for hyperactivity/impulsivity (see *Method* section of the manuscript). [2]The family adversity index was identical in models at 6-7 years and models at 8-12 years; it was based on data collected when the child was 6 years and we used the imputed variable, see the *Method* section of the manuscript. ***p.<.001; **p.<.01; *p.<.05; †p.<.10.

Table S3: Prediction of Males' Non-Violent, Drug-related, Violent and Mixed Court Records (Teachers' ratings)

	Non-violent		Drug-related		Violent		Mixed	
Adolescence	OR	(95% CI)	OR	(95% CI)	OR	(95% CI)	OR	(95% CI)
6-7 years								
Inattention	1.03	0.94-1.14	0.97	0.78-1.21	1.01	0.81-1.26	1.03	0.90-1.17
Hyperactivity[1]	0.98	0.81-1.20	1.01	0.66-1.55	1.10	0.71-1.70	1.09	0.84-1.41
Physical aggression	1.23**	1.06-1.43	1.31[†]	0.97-1.79	1.25	0.91-1.71	1.40***	1.17-1.69
Family adversity[2]	4.47***	2.18-9.19	1.07	0.21-5.47	8.86**	1.83-43.04	7.43***	2.89-19.15
8-12 years								
Inattention	1.08	0.97-1.22	0.76*	0.57-1.00	1.08	0.84-1.40	1.11	0.95-1.30
Hyperactivity/Impulsivity [1]	1.03	0.90-1.17	1.32[†]	0.99-1.75	0.75[†]	0.55-1.03	0.97	0.82-1.15
Physical aggression	1.33**	1.09-1.62	1.20	0.78-1.84	2.32***	1.59-3.36	1.86***	1.47-2.36
Family adversity[2]	4.07***	1.97-8.39	1.31	0.26-6.66	9.60***	1.86-49.44	7.78***	2.92-20.70
Adulthood								
6-7 years								
Inattention	0.98	0.88-1.08	0.96	0.76-1.22	1.19	0.92-1.54	0.93	0.80-1.09
Hyperactivity[1]	0.87	0.70-1.07	1.07	0.68-1.68	1.07	0.65-1.77	1.06	0.79-1.43
Physical aggression	1.47***	1.27-1.70	1.28	0.92-1.79	1.46*	1.05-2.05	1.51***	1.23-1.87
Family adversity[2]	3.26**	1.56-6.81	1.97	0.37-10.49	1.83	0.29-11.6	5.85**	2.00-17.14
8-12 years								
Inattention	1.09	0.97-1.23	0.88	0.66-1.16	1.21	0.89-1.64	1.05	0.87-1.27
Hyperactivity/Impulsivity [1]	1.01	0.89-1.16	1.26	0.94-1.69	0.98	0.71-1.35	1.11	0.93-1.34
Physical aggression	1.23*	1.00-1.50	0.98	0.60-1.61	1.63*	1.05-2.54	1.71***	1.31-2.22
Family adversity[2]	3.00**	1.44-6.25	2.25	0.43-11.9	2.16	0.31-14.9	5.25**	1.71-16.05

Note. The table presents Odds ratios (OR) and 95% Confidence intervals (CI) of multinomial logistic regressions predicting males criminal records. Teacher's ratings were available for all male participants at age 6-7 years (N = 1398) and missing for 16 participants at 8-12 (N = 1382). [1]Assessments of hyperactivity at 6-7 years were based on two items; at 8-12 years, 5 items were available for hyperactivity/impulsivity (see method section). [2]The family adversity index was identical in models at 6-7 years and models at 8-12 years; it was based on data collected when the child was 6 years and we used the imputed variable, see the *Method* section of the manuscript. [***]p.<.001; [**]p.<.01; [*]p.<.05; [†]p.<.10.

Table S4: Prediction of Males' Non-Violent, Drug-related, Violent and Mixed Court Records (Mothers' ratings)

	Non-violent		Drug-related		Violent		Mixed	
Adolescence	OR	(95% CI)	OR	(95% CI)	OR	(95% CI)	OR	(95% CI)
6-7 years								
Inattention	0.99	0.88-1.12	0.89	0.68-1.16	1.25	0.95-1.64	1.01	0.86-1.19
Hyperactivity[1]	1.07	0.89-1.29	1.04	0.70-1.55	0.76	0.48-1.20	1.01	0.78-1.31
Physical aggression	1.23*	1.04-1.46	1.25	0.87-1.81	1.91***	1.34-2.72	1.46***	1.17-1.82
Family adversity[2]	4.62***	2.23-9.58	1.24	0.24-6.43	6.42*	1.26-32.66	7.21***	2.75-18.88
8-12 years								
Inattention	1.00	0.88-1.14	0.90	0.66-1.21	0.91	0.66-1.25	0.89	0.73-1.09
Hyperactivity/Impulsivity [1]	1.07	0.95-1.22	1.20	0.91-1.58	1.04	0.78-1.40	1.17†	0.98-1.40
Physical aggression	1.25*	1.01-1.54	1.03	0.63-1.66	1.78*	1.14-2.79	1.56**	1.18-2.06
Family adversity[2]	4.28***	2.05-8.97	1.30	0.23-7.51	7.94*	1.47-42.98	8.05***	2.82-23.03
Adulthood								
6-7 years								
Inattention	1.04	0.92-1.18	1.12	0.85-1.49	1.21	0.90-1.63	0.98	0.82-1.19
Hyperactivity[1]	0.94	0.77-1.14	0.65†	0.41-1.04	1.25	0.78-2.02	1.40*	1.04-1.90
Physical aggression	1.33**	1.12-1.58	1.50*	1.02-2.22	0.90	0.57-1.41	1.33*	1.03-1.71
Family adversity[2]	3.24**	1.55-6.78	2.36	0.42-13.33	2.39	0.36-15.81	4.04*	1.33-12.34
8-12 years								
Inattention	1.03	0.90-1.18	1.12	0.82-1.51	0.98	0.68-1.41	0.99	0.80-1.22
Hyperactivity/Impulsivity [1]	1.11†	0.98-1.27	0.92	0.68-1.24	1.47*	1.06-2.05	1.20†	0.99-1.46
Physical aggression	1.27*	1.03-1.57	1.42	0.87-2.30	0.81	0.45-1.46	1.46*	1.08-1.98
Family adversity[2]	3.73***	1.74-7.97	2.40	0.41-14.14	1.87	0.23-15.47	7.73***	2.48-24.05

Note. The table presents Odds ratios (OR) and 95% Confidence intervals (CI) of multinomial logistic regressions predicting males criminal records. Mothers' ratings were missing for 8 male participants at age 6-7 years (N = 1390) and missing for 109 participants at 8-12 (N = 1289). [1]Assessments of hyperactivity at 6-7 years were based on two items; at 8-12 years, 5 items were available for hyperactivity/impulsivity (see method section). [2]The family adversity index was identical in models at 6-7 years and models at 8-12 years; it was based on data collected when the child was 6 years and we used the imputed variable, see the *Method* section of the manuscript. ***p.<.001; **p.<.01; *p.<.05; †p.<.10

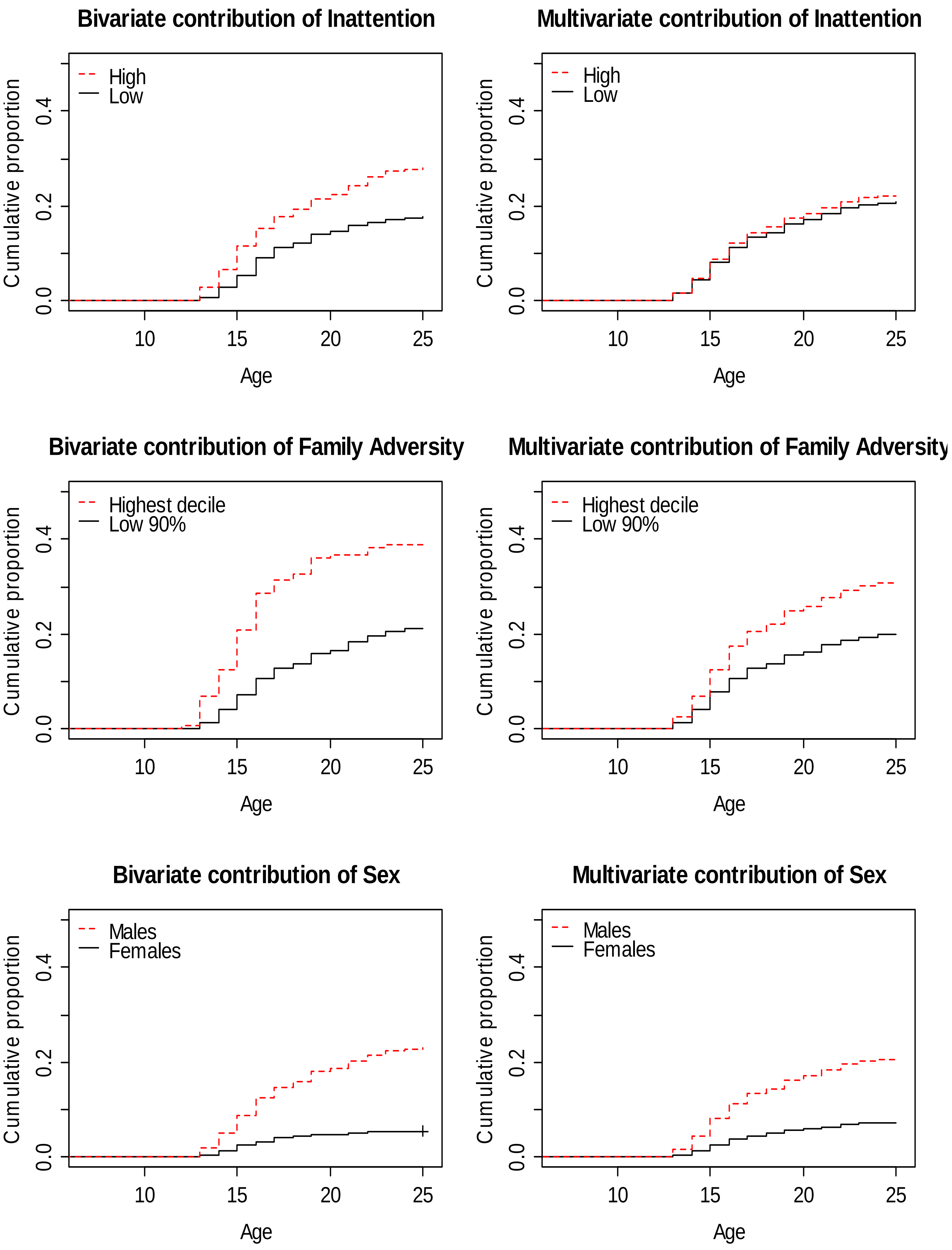
Bivariate contribution of Inattention
High
Low
Multivariate contribution of Inattention
High
Low
Bivariate contribution of Family Adversity
Highest decile
Low 90%
Multivariate contribution of Family Adversity
Highest decile
Low 90%
Bivariate contribution of Sex
Males
Females
Multivariate contribution of Sex
Males
Females
Cumulative proportion
0.0
0.2
0.4
10
15
20
25
Age

Selection of trajectories

To determine the number of trajectories we used an indice derived from Calinski & Harabatz. Below is a graph plotting the values of the indice for different potential solutions (from 2 up to 6 trajectories). Regarding inattention, a two trajectories solution was the best: the indice decreased as the number of trajectories increased. Regarding physical aggression there was a substantial rise from two to three trajectories, then a stagnation followed by a clear decrease from five to six. Although the indice for the solution with five trajectories was slightly better than for the solution with only four, the fifth trajectory did not add any clinically relevant information (i.e. splitting one trajectory into two parallel ones). We finally retained the four trajectories solution. The situation was similar for the hyperactivity trajectories and we also selected the four trajectories solution.

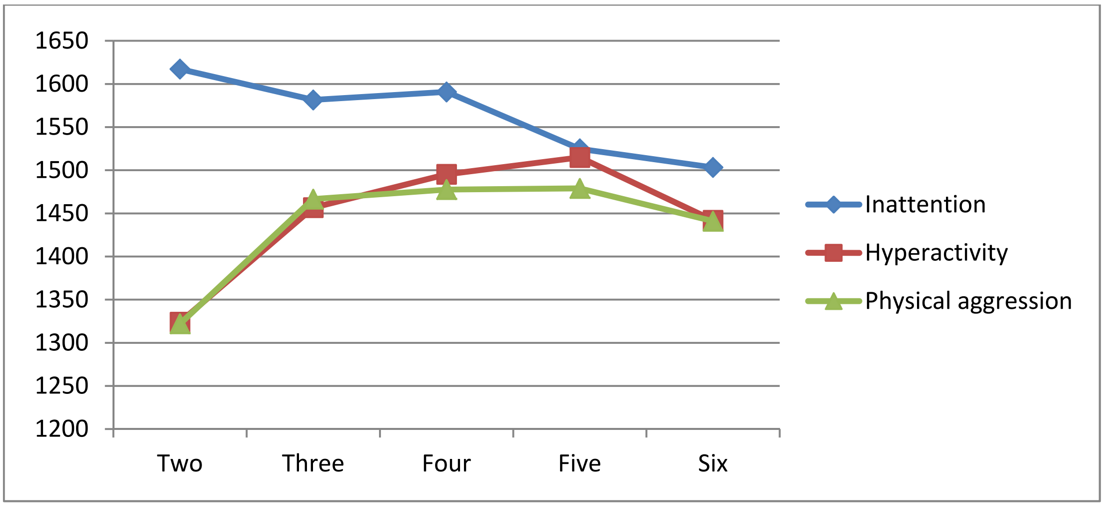

Representation of trajectories

We propose two graphs to represent the trajectories for each behavior. First, a two dimensional representation of the joint trajectories clustering with mother and teacher rated levels as well as the percentages of participants in each trajectory; second, a three dimensional dynamic graph which can be manipulated by the viewer (using the computer mouse). Trajectories' colors remain the same in the two graphs. X is the time, Z corresponds to mother ratings whereas Y corresponds to teacher ratings. It is to be noted that mother and teacher rated behavioral levels in the first graph are a projection of the three dimensional trajectories on, respectively, the Z and the Y plans.

Joint trajectories of inattention (2d representation)

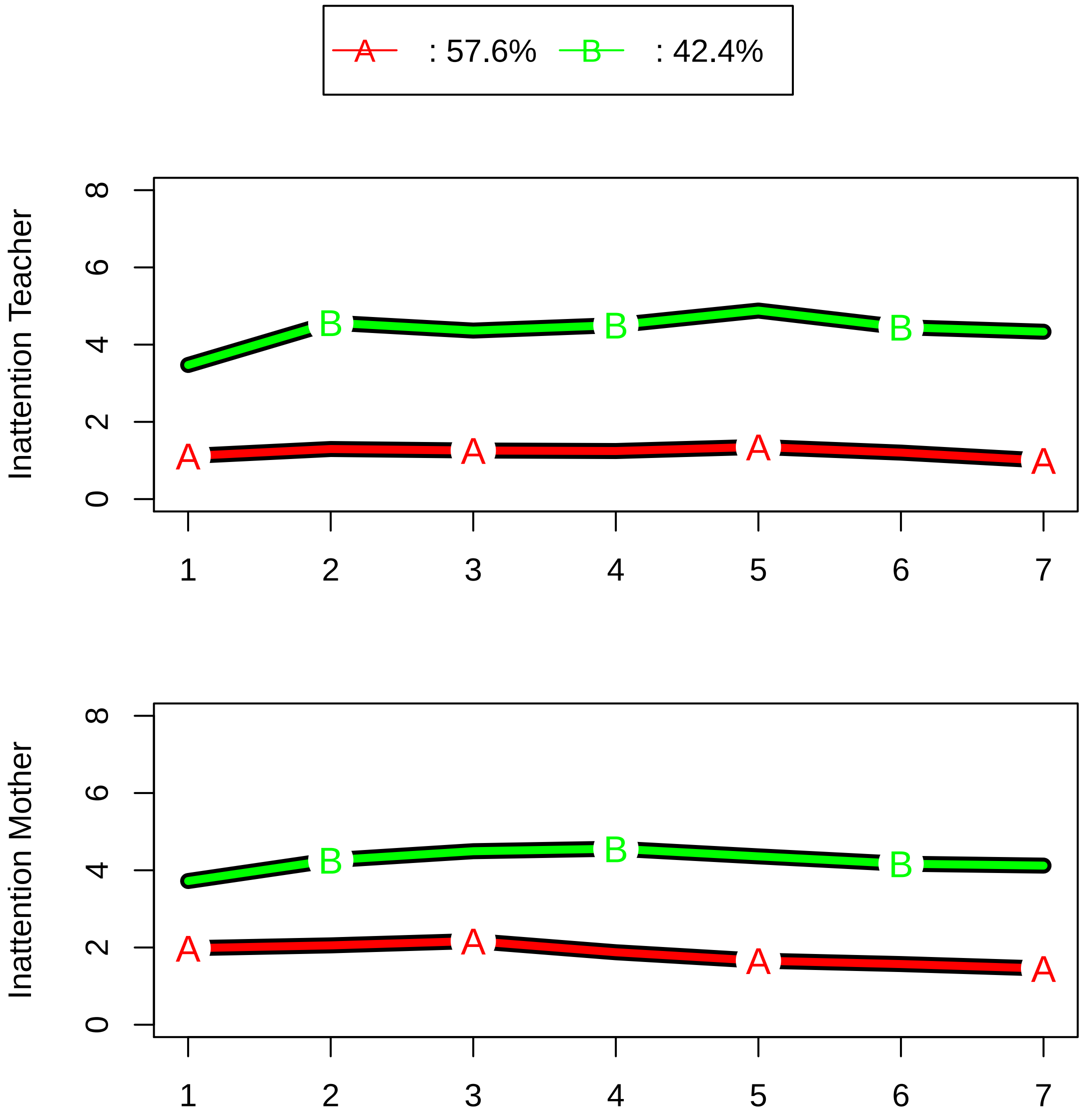

Joint trajectories of Inattention (3d representation)

X: Time
Z: Mothers' ratings
Y: Teachers' ratings

Joint trajectories of hyperactivity (2d representation)

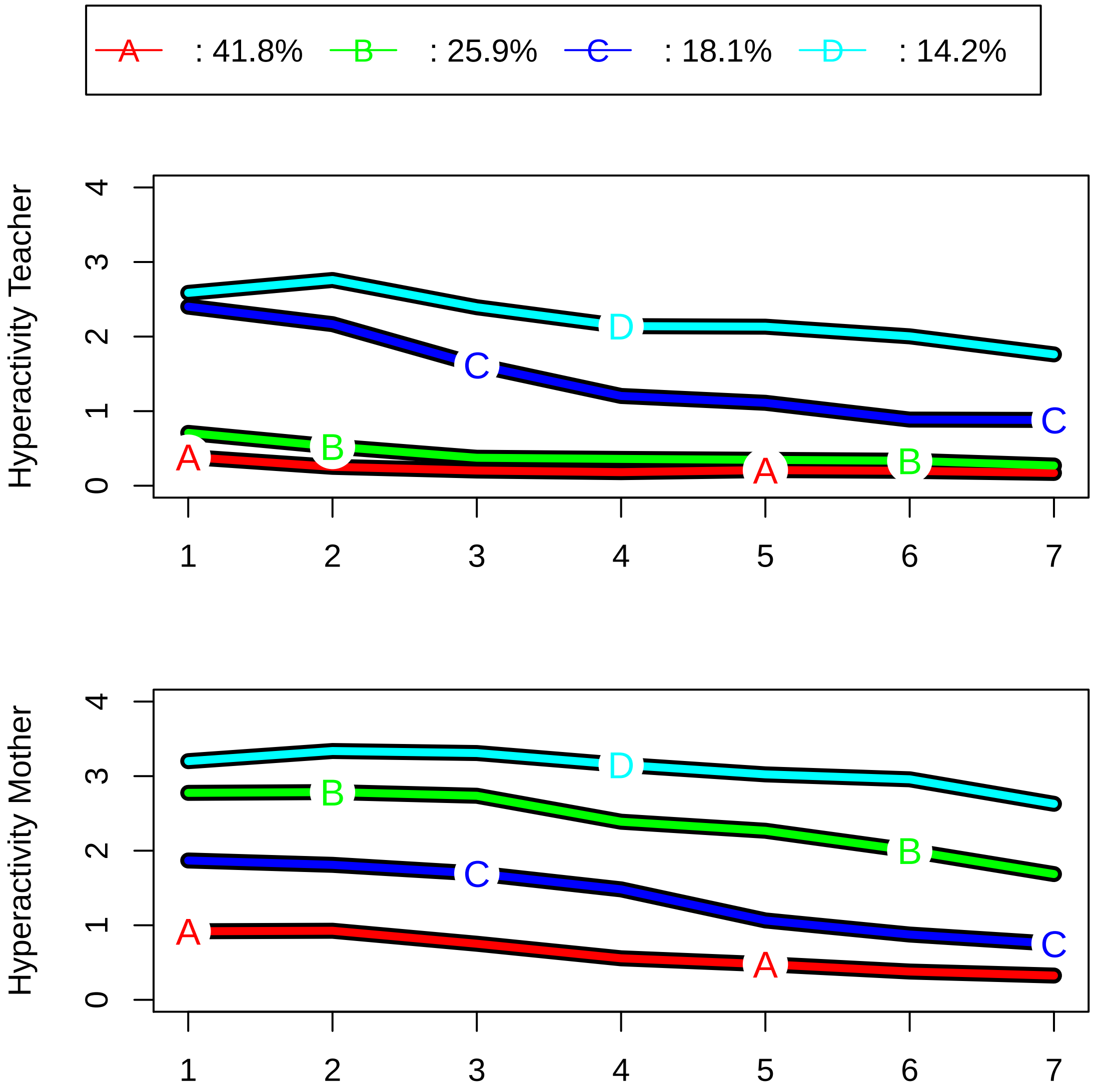

Joint trajectories of Hyperactivity (3d representation)

X: Time
Z: Mothers' ratings
Y: Teachers' ratings

Joint trajectories of Physical aggression (2d representation)

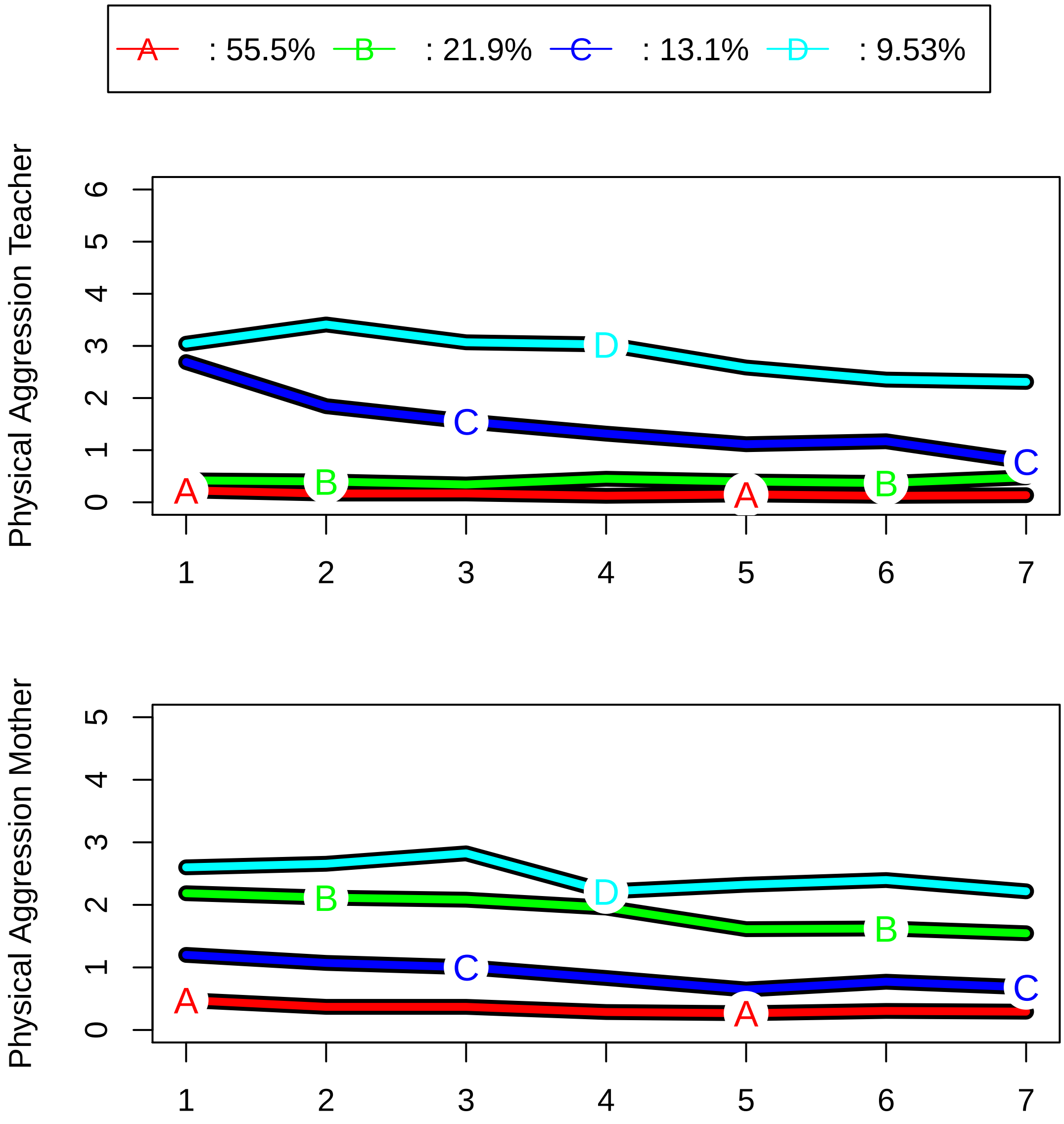

Joint trajectories of Physical Aggression (3d representation)

X: Time
Z: Mothers' ratings
Y: Teachers' ratings
